# Supplementary figures and images for: Aberrant subchondral osteoblastic metabolism modifies NaV1.8 for osteoarthritis
Source: eLife. 2020 May 22;9:e57656. doi: 10.7554/eLife.57656 (PMC7308086; doi:10.7554/eLife.57656)

Full scan of western blots in Figure 1f

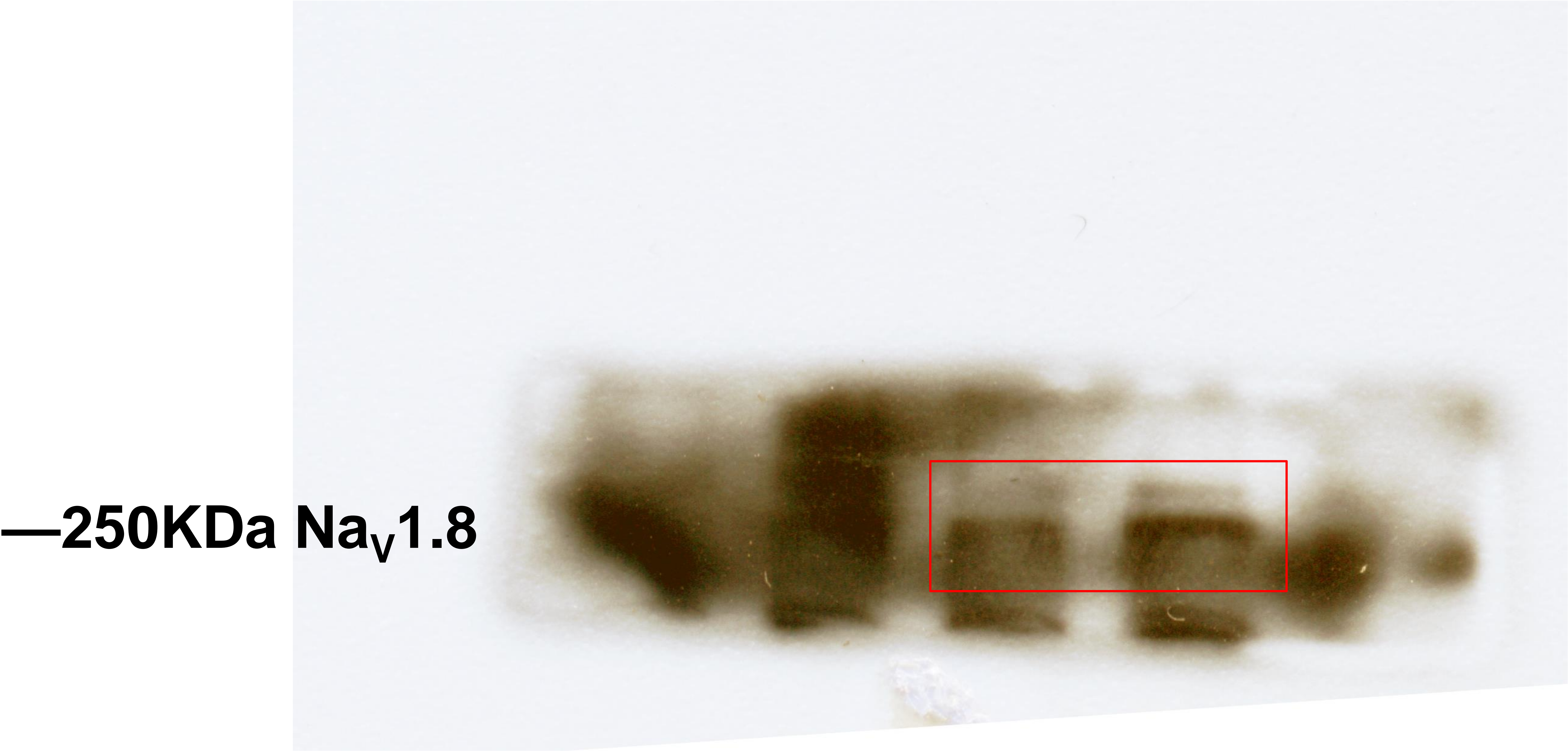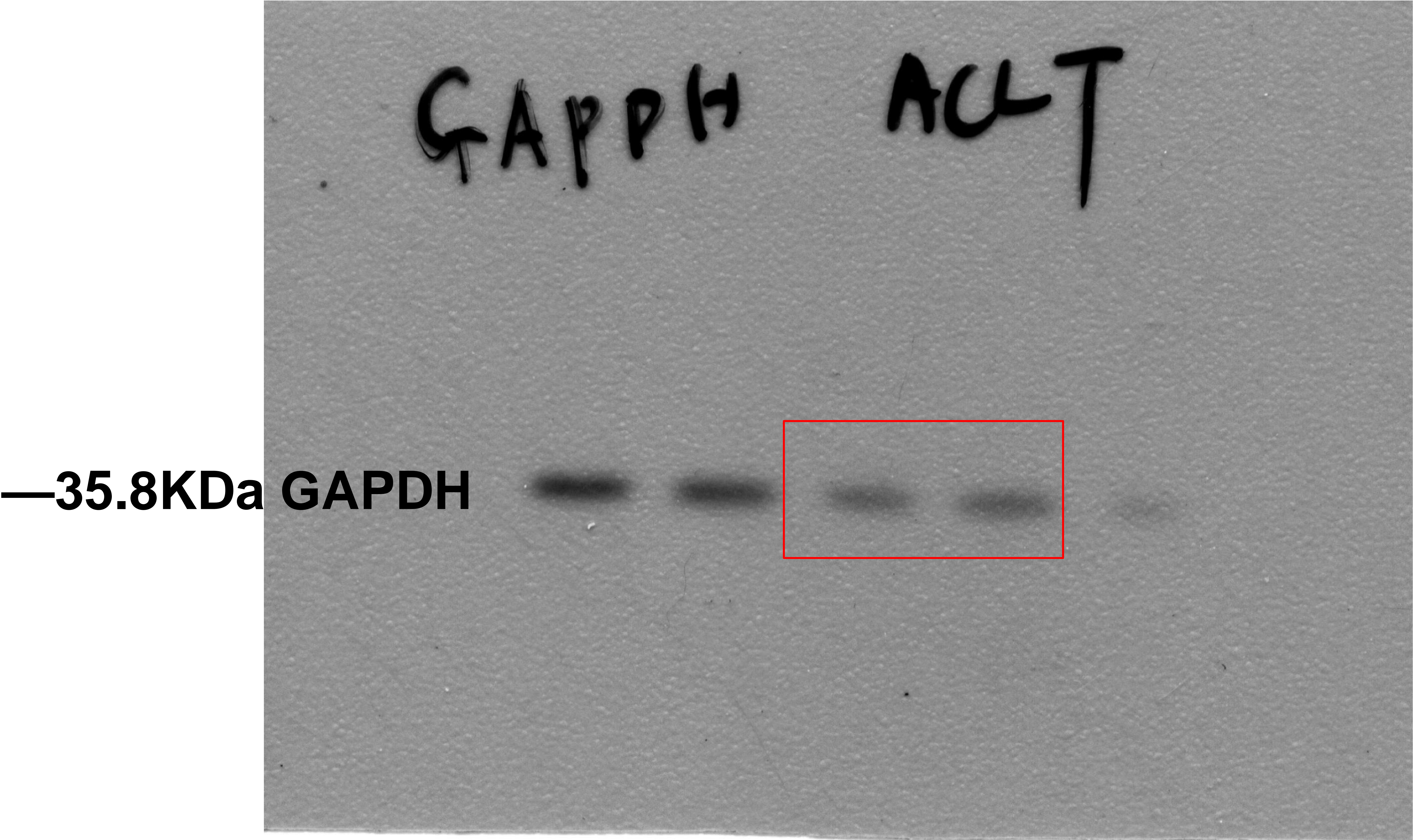

Supplement: Figure 1—source data 2. [file elife-57656-fig1-data2.pdf]

Full scan of western blots in Figure 2 figure Supplement 1i

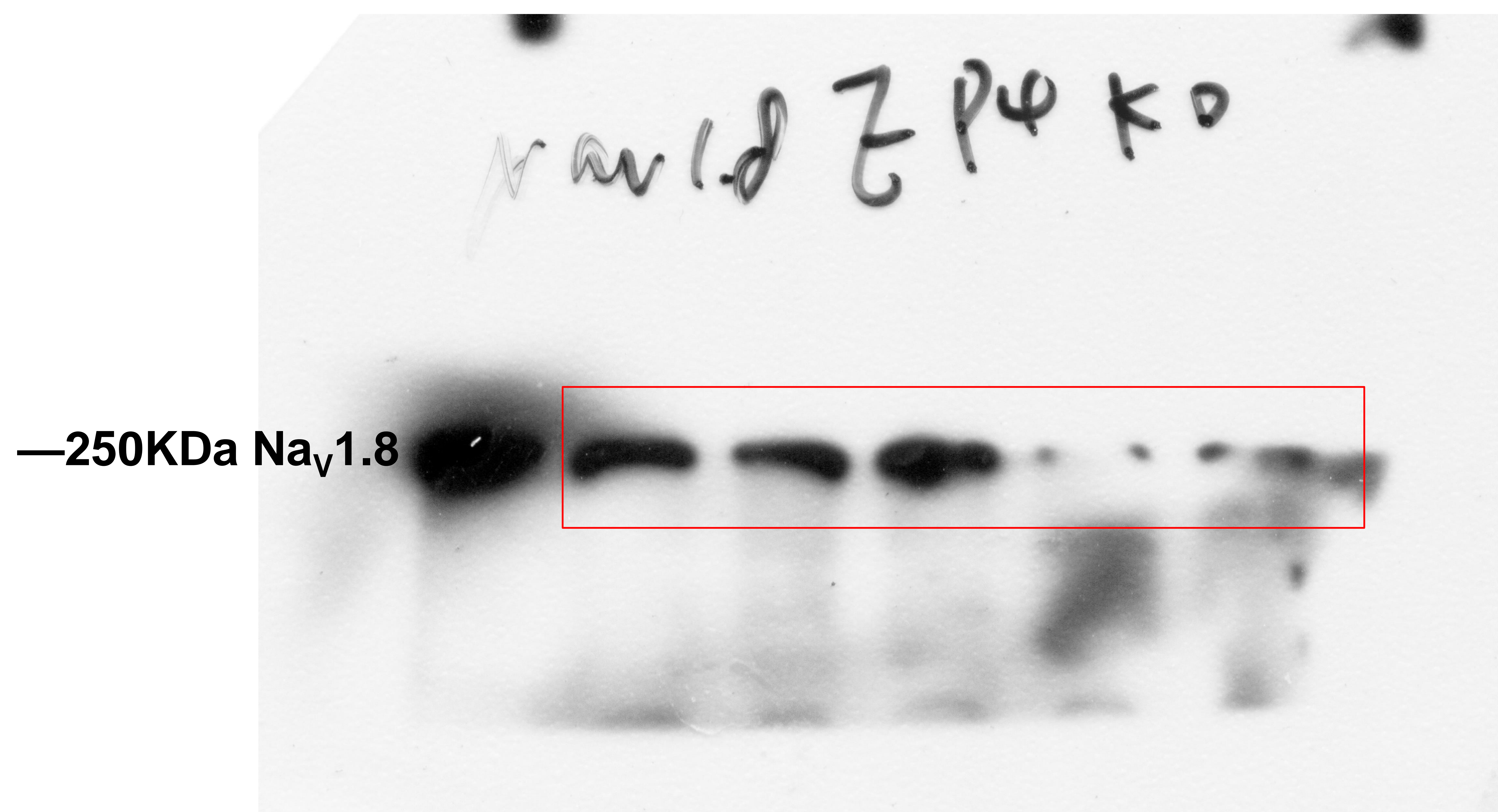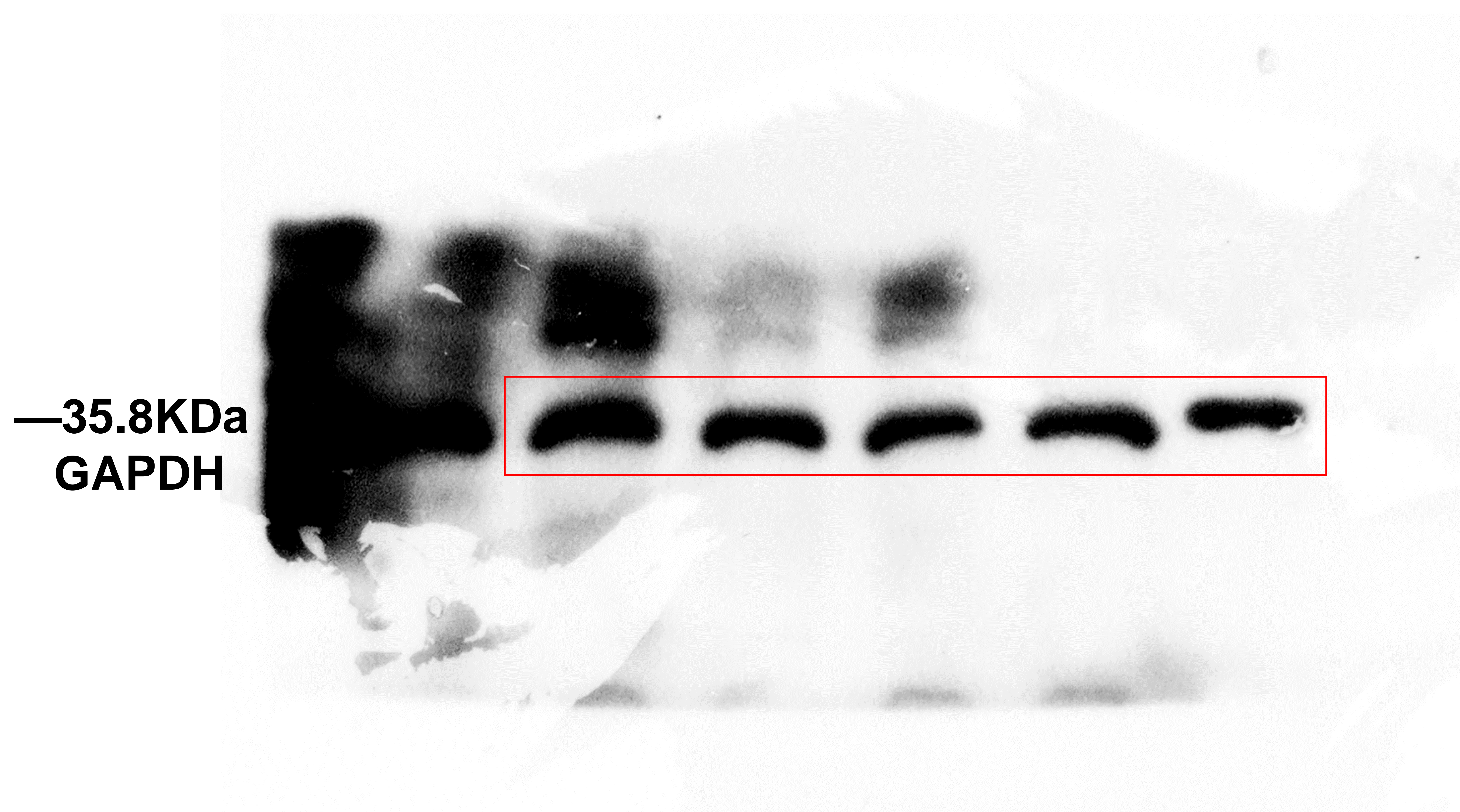

Supplement: Figure 2—figure supplement 1—source data 1. [file elife-57656-fig2-figsupp1-data1.pdf]

Full scan of western blots in Figure 4b

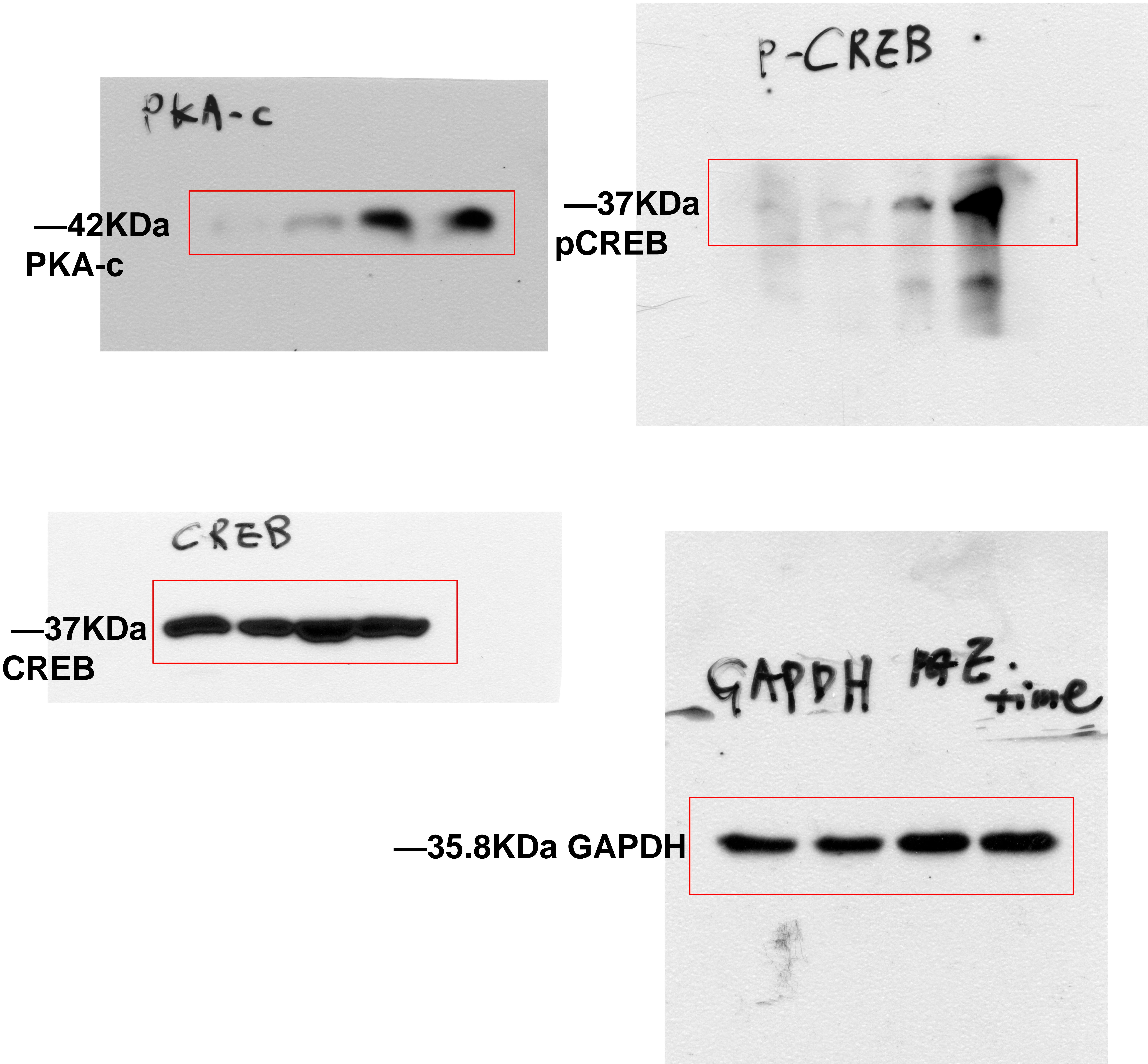

Supplement: Figure 4—source data 2. [file elife-57656-fig4-data2.pdf]

Full scan of western blots in Figure 4c

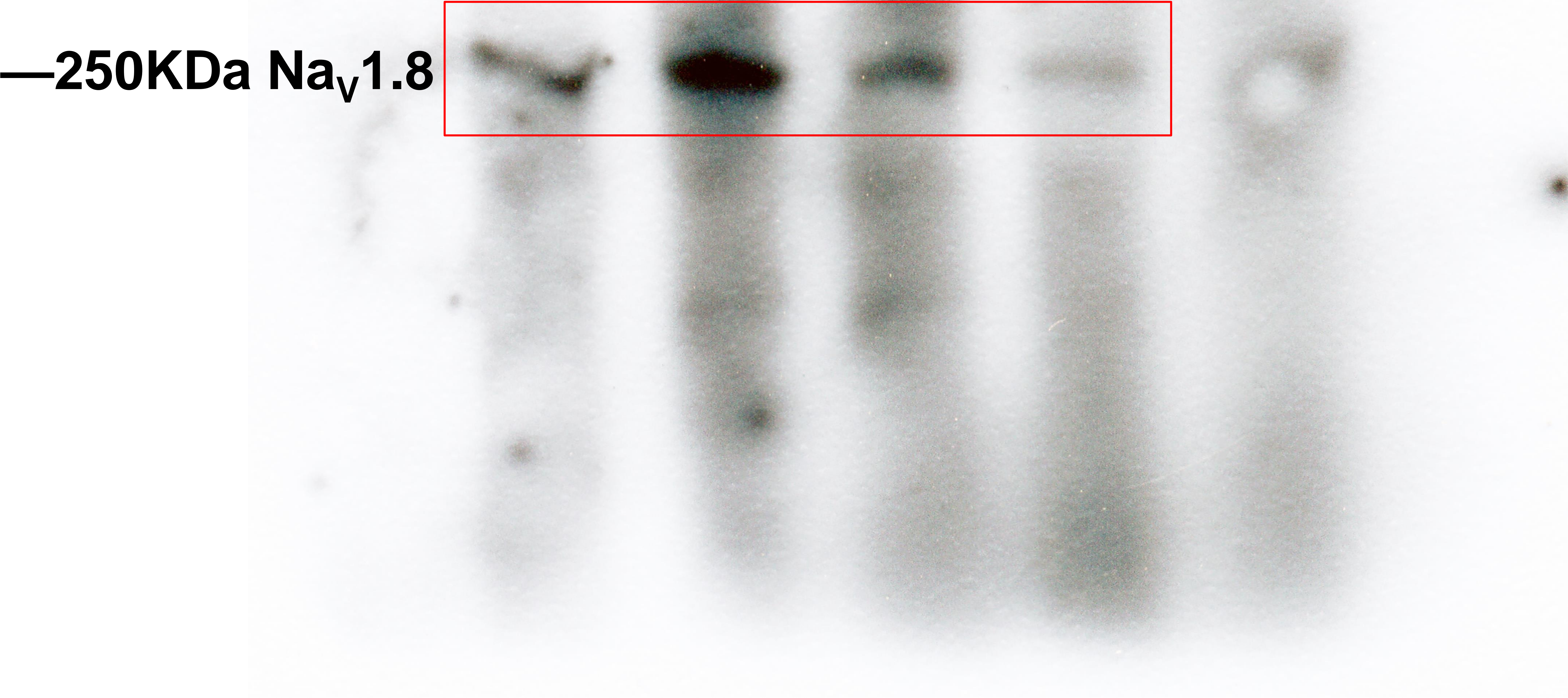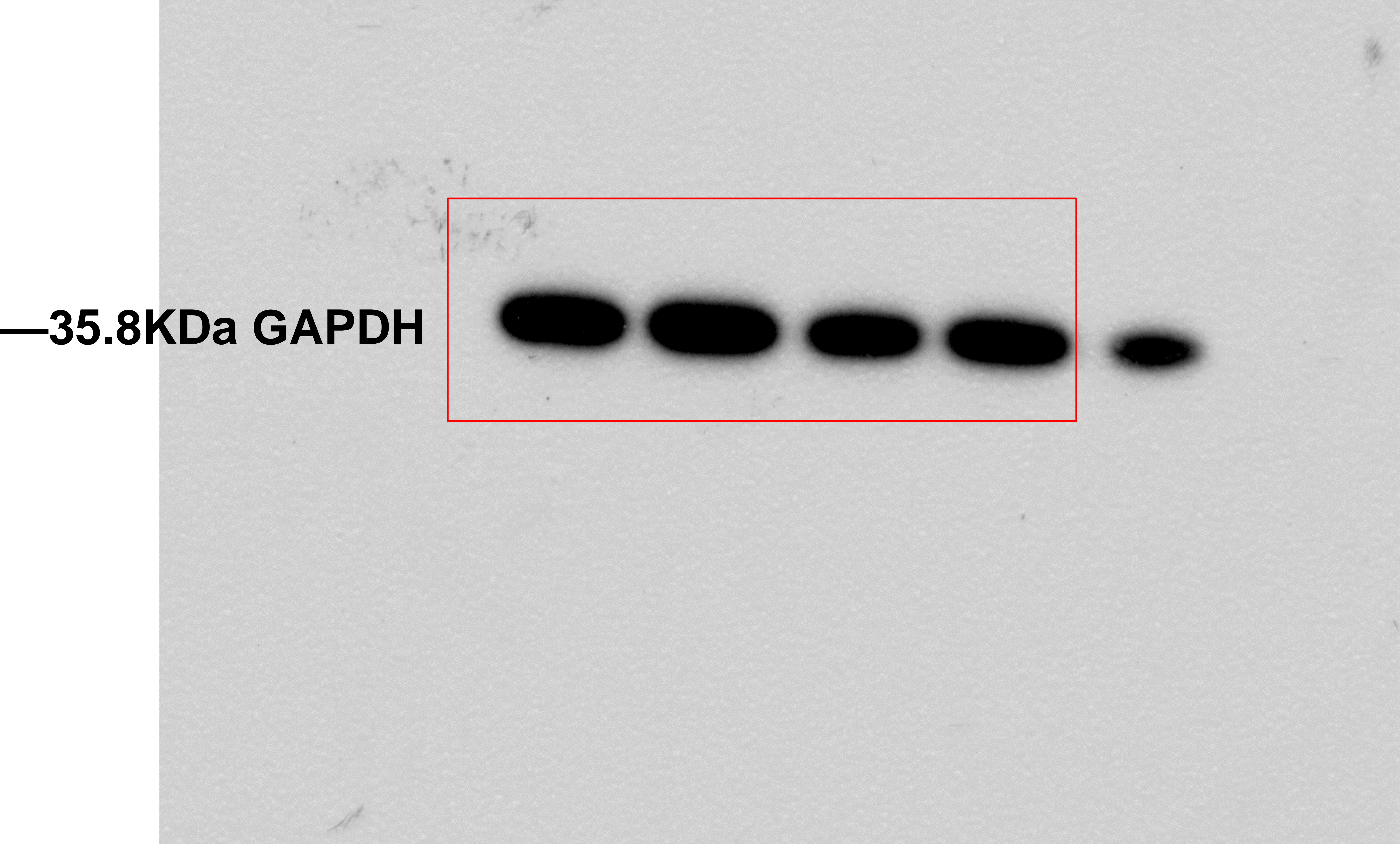

Supplement: Figure 4—source data 3. [file elife-57656-fig4-data3.pdf]

Full scan of DNA gel in Figure 4k

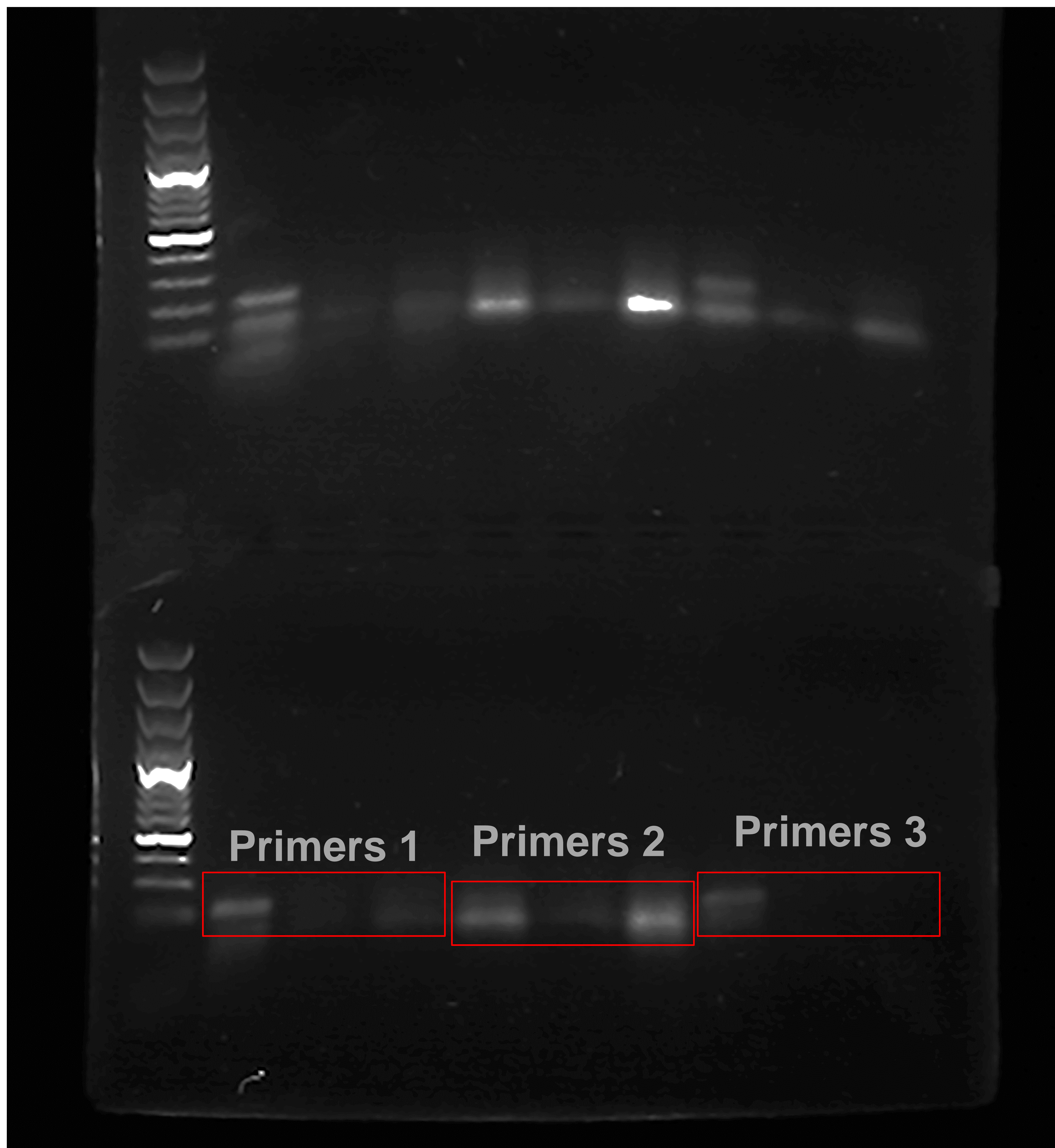

Supplement: Figure 4—source data 4. [file elife-57656-fig4-data4.pdf]

Full scan of western blots in figure 6m

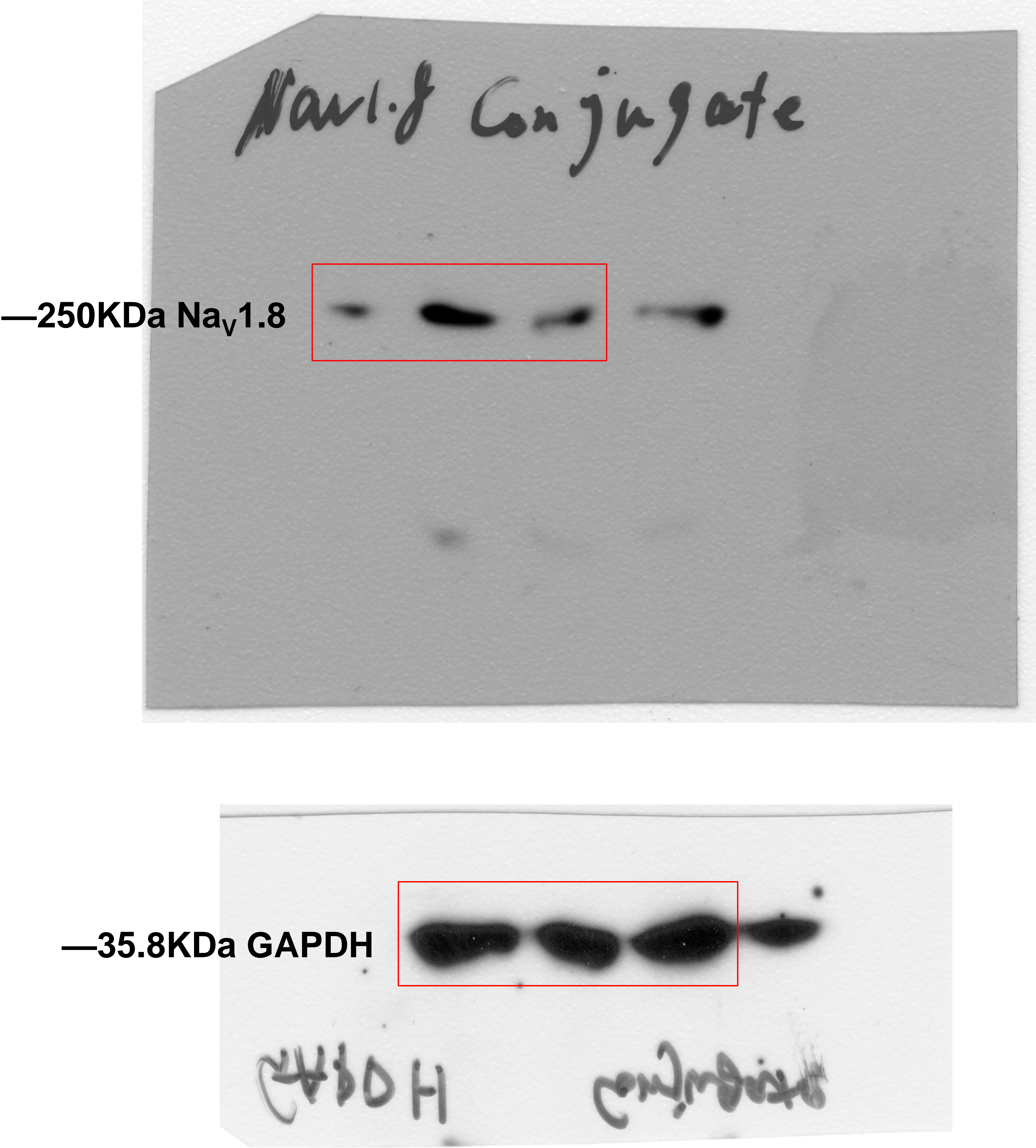

Supplement: Figure 6—source data 2. [file elife-57656-fig6-data2.pdf]
